# German Translation and Validation of the “Freezing of Gait Questionnaire” in Patients with Parkinson's Disease

**DOI:** 10.1155/2015/982058

**Published:** 2015-01-29

**Authors:** Anina Vogler, Jorina Janssens, Thomas Nyffeler, Stephan Bohlhalter, Tim Vanbellingen

**Affiliations:** ^1^Health, Bern University of Applied Sciences, 3008 Bern, Switzerland; ^2^Neurorehabilitation Centre Klinik Bethesda Tschugg, 3233 Tschugg, Switzerland; ^3^Neurology and Neurorehabilitation Centre, Luzerner Kantonsspital, 6000 Luzern, Switzerland; ^4^University of Bern, 3010 Bern, Switzerland

## Abstract

*Background*. Freezing of Gait (FOG) is a disabling parkinsonian symptom. The Freezing of Gait Questionnaire (FOG-Q) reliably detects FOG in patients with Parkinson's disease (PD). *Objectives*. The aim of this study was to develop a German translated version of the FOG-Q and to assess its validity. *Methods*. The translation was accomplished using forward-backward-translation. The construct validity of the FOG-Q was examined in twenty-seven German native speaking PD patients. Convergent validity was assessed by correlating the FOG-Q with the Movement Disorder Society-Unified Parkinson's Disease Rating Scale (MDS-UPDRS) II-III, the Parkinson Disease Questionnaire 39 (PDQ-39), and the Timed Up and Go Test (TUG). Divergent validity was assessed by correlating the FOG-Q with the MDS-UPDRS I. The internal consistency was measured using Cronbach's alpha (C*α*). *Results*. A good internal structure of the FOG-Q was found (C*α* = 0.83). Significant moderate correlations between the FOG-Q and the MDS-UPDRS item 2.13 (freezing) (*r*_*s*_ = 0.568, *P* = 0.002) and between the FOG-Q and the PDQ-39 subscale mobility (*r*_*s*_ = 0.516, *P* = 0.006) were found. The lack of correlation with the MDS-UPDRS I demonstrated good divergent validity. *Conclusion*. The German FOG-Q is a valid tool to assess FOG in German native speaking PD patients.

## 1. Introduction

Parkinson's disease (PD) is one of the most common neurodegenerative diseases [1] mainly characterized by a progressive degeneration of dopaminergic neurons in the substantia nigra, which belongs to the basal ganglia [2]. A frequently observed symptom in advanced stages of PD is Freezing of Gait (FOG) [3]. The prevalence of FOG lies between 20 and 60% [4]. This disabling clinical phenomenon is defined as follows: “brief, episodic absence or marked reduction of forward progression of the feet despite the intention to walk” [5]. Patients often describe FOG as “having the feeling as if their feet are glued to the ground.” It is often triggered by stressful situations, for example, when walking in crowded places or walking through narrow spaces, such as crossing the doorstep [6]. Furthermore, FOG is often associated with falls [7] and reduced quality of life (QoL) [8]. Therefore, most patients who experience FOG describe the symptom as very disruptive during the performance of several activities of daily living (ADL) [9].

FOG may improve with increased attention in the wide spaces of the therapeutic setting [10]. For this reason, standardized information on the frequency and severity of FOG is important. Several assessments have been developed so far to quantify FOG [11–16]. However, only the Freezing of Gait Questionnaire (FOG-Q), the New Freezing of Gait Questionnaire (NFOG-Q), and the self-administered version of the FOG-Q (FOG-Qsa) assess FOG from a patient's perspective [11, 12, 14].

The FOG-Q is a well-validated and worldwide used measurement tool [6, 11, 17, 18]. There are recommendations, concerning the translation and cross-cultural adaption of measurements, which indicate the importance of a comprehensive translation procedure [19]. This procedure respects cross-cultural adaptations and linguistic differences between the original measurement and the newly translated one. Furthermore, it is recommended that psychometric properties should be redefined [19].

The aim of this study is to develop a German translated version of the FOG-Q, which is valid to assess FOG in patients with PD. We hypothesised that the translated FOG-Q correlated significantly with items 2.13 (freezing) and 3.11 (FOG) of the Movement Disorders Society-Unified Parkinson's Disease Rating Scale (MDS-UPDRS), which is the gold standard to assess Parkinsonian symptoms [20]. Furthermore we predicted significant correlations with the Timed Up and Go Test (TUG), a sensitive test to provoke FOG [21], and the Parkinson's Disease Questionnaire 39 (PDQ-39), which measures QoL with respect to mobility and ADL. In contrast we expected no correlation with the MDS-UPDRS subscale I, which assessed general cognitive aspects of PD, because in this study only patients with mild or no cognitive impairments were included.

## 2. Material and Methods

### 2.1. Subjects

Twenty-seven German-speaking patients with Parkinsonism were recruited from two neurorehabilitation centres (Klinik Bethesda, Tschugg, Switzerland, and Luzerner Kantonsspital, Luzern, Switzerland), a physiotherapy practice (Robellaz physiotherapy & training GmbH, Köniz, Switzerland), and a medical office (Neurozentrum Bern, Bern, Switzerland). Twenty-five of them were diagnosed with idiopathic Parkinsonism and two with atypical Parkinsonism. Diagnosis was done by expert neurologists according to the criteria of the United Kingdom Brain Bank [22]. Further inclusion criteria were the presence of FOG, described by the patient, during the week before the actual measurements of the present study, and a score above 21/30 on the Montreal Cognitive Assessment (MoCA). Clinical characteristics of patients are summarized in Table 1.

The study was conducted according to the ethical principles of the declaration of Helsinki (1975) and was approved by the Cantonal Ethics Committee of Bern (KEK). Written informed consent was obtained from all patients.

### 2.2. Material

The FOG-Q consists of six questions [17]. Questions 1, 2, 4, 5, and 6 refer to the patient's experiences, related to FOG, of the previous week. For question 3 the patient is asked about his unique experience of FOG in different situations, which is not limited in time. Each question has a 5-point scale, where 0 means an absence of symptoms and 4 represents the worst stage [11]. Consequently, the total score on the FOG-Q ranges from 0 to 24 points. The higher the score is, the more the FOG is pronounced. The time needed to administer the questionnaire is approximately 5–10 minutes.

The German version of the FOG-Q (the appendix) was established by the authors based on a forward-backward-translation according to Beaton et al. [19].

The MDS-UPDRS is divided into four subscales: subscale I (nonmotor experiences of daily living), subscale II (motor experiences of daily living), and subscale IV (motor complications) are patient- and caregiver-oriented questionnaires. Instead, part III (motor examination) is an objective assessment of the patient's motor abilities. Each question has a 5-point scale, where 0 means an absence of symptoms and 4 represents the worst stage.

The PDQ-39 is a subjective questionnaire to assess QoL in patients with PD [23]. The questionnaire consists of thirty-nine questions, which are divided to eight subscales (mobility, ADL, emotional well-being, stigma, social support, cognitions, communication, and bodily discomfort). Each question has a range from 0 (no problem at all) to 100 (maximum level of problem).

The TUG is a measurement tool to judge PD patients' mobility while rising from an arm chair, walking three meters, turning, walking back, and sitting again [24, 25]. Patients' performance is measured in seconds.

### 2.3. Procedures

In this cross sectional pilot study each patient was measured at only one point in time. All assessments were performed during the ON state, when the dopaminergic drug effects are at their peak and patients are in their best corporal agility. All assessments were carried out in a standardized order by one author (Anina Vogler).

First, the FOG-Q was filled in by the author, who asked the patient each question and explained or demonstrated FOG if necessary [11]. Subsequently, the TUG was assessed followed by the MDS-UPDRS subscales I–III. Finally the PDQ-39 was conducted.

To further improve reliability of rating, patient's performance on the MDS-UPDRS subscales I–III and the TUG was videotaped. Afterwards a blinded investigator (Jorina Janssens) rated the videos.

### 2.4. Statistical Analyses

The statistics were performed using the Statistical Package for the Social Sciences (SPSS) (Version 21; SPSS IBM, NY, U.S.A).

The first objective of the statistical analyses was to measure the internal consistency of the total FOG-Q by using Cronbach's alpha (C*α*). A value above 0.80 is an indicator of a good homogeneity of items within the total scale [26].

Furthermore, the construct validity, which incorporates convergent and divergent validity, of the FOG-Q was examined. Convergent validity was assessed by examining the pattern of Spearman's rho (*r*_*s*_) correlations, which is used for ordinal data, between the FOG-Q and the MDS-UPDRS items 2.13 and 3.11, the TUG and the PDQ-39 subsections mobility, and ADL. Divergent validity was assessed by examining the correlation between the FOG-Q and the MDS-UPDRS subscale I.

The two-sided level of significance was *P* < 0.05.

## 3. Results

The total FOG-Q score ranged between 7 and 21 points with a mean of 13.89 (SD ± 3.555). The mean FOG-Q item scores ranged between 1.89 and 2.70 (SD ± 0.465–1.014).

The statistical analysis revealed a good internal consistency (C*α* = 0.83). This result indicated a good internal reliability of the FOG-Q.

An overview of Spearman's correlations demonstrating convergent and divergent validity is shown in Table 2.

These results showed no association between the FOG-Q and the MDS-UPDRS subscale I (*r*_*s*_ = 0.344, *P* = 0.079), indicating a good divergent validity.

By contrast, significant correlations between the FOG-Q and the MDS-UPRS item 2.13 (*r*_*s*_ = 0.568, *P* = 0.002) and the subscales mobility (*r*_*s*_ = 0.516, *P* = 0.006) and ADL (*r*_*s*_ = 0.407, *P* = 0.035) of the PDQ-39 demonstrated good convergent validity.

## 4. Discussion

The FOG-Q has been chosen for the translation into German and its validation for different reasons. First, the FOG-Q is a well-known and often used measurement tool in clinical settings. Second, the FOG-Q is easy and short to administer, in contrast to the more newly developed NFOG-Q, which requires video monitoring. Third, the FOG-Q has already shown highly reliable and valid detection of FOG in PD patients in previous studies [6, 11, 17, 18].

In the present study we demonstrated a good internal consistency (C*α* = 0.83) of the German FOG-Q, a value that is comparable with the values found in previous validation studies [6, 11, 17, 18]. Furthermore we showed good construct validity of the German FOG-Q indicated by significant correlations with the MDS-UPDRS item 2.13 and the PDQ-39 subsections mobility and ADL. Our results correspond with findings from previous validation studies [6, 11, 17, 18]. The significant correlation between the FOG-Q with the subscales of the PDQ-39 indicates that patients with more pronounced FOG state more problems in QoL [8]. This finding underlines the relevance of using the FOG-Q as a measurement tool to assess therapeutic effects, which are expected when PD patients are specifically treated to overcome FOG in different ADL.

In contrast to the Swedish validation study [6], we did not find significant correlations between the FOG-Q and the MDS-UPDRS total subscales II and III and the TUG. A possible reason could be that, except for MDS-UPDRS items 2.13 and 3.11, none of the other items of subscales II and III of the MDS-UPDRS refers directly to FOG. Furthermore, we could also not find a significant correlation between the German FOG-Q and the MDS-UPDRS item 3.11. Since FOG is more pronounced in daily situations and can be differently expressed depending on the motor state (ON/OFF) [11], the measurement in the ON state and the therapeutic setting could have influenced the results of the motor assessments (MDS-UPDRS item 3.11 and TUG).

Some patients described difficulties to exactly estimate the occurrence of FOG, as well as its duration. Sometimes the FOG episodes showed a different picture than the subjective self-assessment of the patients. In this case, assessors must avoid influencing patients' answers, since it is a subjective measurement tool. This uncertainty of patient's perception about FOG and their difficulty to differ FOG from OFF akinesia were already described previously [17]. Akinesia means a lack of movement that is not caused by paralysis [27]. Although the NFOG-Q improved these limitations, the final recommendation was not to use the NFOG-Q for routine clinical assessments [12]. The authors mentioned some impracticability, related to the fact that video analysis is required [12].

In line with previous validation studies [6, 11, 18], we also included only PD patients without dementia. The reason is that due to reduced cognitive abilities of PD patients with dementia the FOG-Q is often difficult to perform since it requires a proper self-perception of the patient. Evidence is accumulating that FOG is not just a pure motor phenomenon but may be caused by motor, affective, and cognitive deficits [28]. Consequently, FOG can be observed more often in more advanced stages of PD [4], which can be related to an increased appearance of cognitive impairments [28]. Therefore, it should be aimed, when identifying and quantifying FOG in the entire PD population, to use a combination of subjective, such as the FOG-Q, and objective measurement tools (MDS-UPDRS item 3.11), which are practicable in daily clinical routine [29]. Recently, another objective approach to classify freezers was developed in which FOG was provoked by letting patients perform several turns while they were in the OFF state [30]. However, a major disadvantage of this approach is that the probability is low to assess FOG in PD patients, in an OFF state, who visit a clinical practice. In addition, the assessment's focus is only on turning and does not address other aspects, such as passing through narrow spaces, which also contribute to FOG.

## 5. Conclusion

The German version of the FOG-Q is a valid tool to assess FOG in PD patients without dementia. It can be used by therapists in the German-speaking parts of Switzerland, in Germany, and in Austria to quantify FOG in PD patients. By now, the FOG-Q appears to be the most appropriate measurement tool to assess FOG in clinical practice, due to its short time to administer and high practicability.

## Figures and Tables

**Table 1:** Clinical characteristics of PD patients (*n* = 27).

Age (y)	68.67 ± 9.17 (45–87)^*^
Sex (m/f)	20/7
MoCA	25.37 ± 2.31 (21–29)^*^
Disease duration (y)	11.26 ± 5.8 (2–26)^*^
Hoehn and Yahr stage (ON)	2.93 ± 0.73 (2–4)^*^
Levodopa equivalent (mg/day)	1,071.96 ± 576 (156–2,793)^*^

y = year; m = male, f = female. ^*^Mean ± SD = standard deviation (range).

**Table 2:** FOG-Q correlations (*n* = 27).

	Correlations (*r*_*s*_)	*P* value
MDS-UPDRS I	0.344	0.079
MDS-UPDRS II	0.247	0.214
MDS-UPDRS III	0.152	0.450
MDS-UPDRS 2.13	0.568	0.002^**^
MDS-UPDRS 3.11	0.118	0.557
PDQ-39 mobility	0.516	0.006^**^
PDQ-39 ADL	0.407	0.035^*^
TUG	0.105	0.604

^**^Significant correlation on the two-sided level of *P* ≤ 0.01.

^*^Significant correlation on the two-sided level of *P* ≤ 0.05.

## Appendix

### Freezing of Gait Questionnaire (German)

*Freezing of Gait Questionnaire (FOG-Q): Deutsche Version*

*Frage 1*. Während Ihres schlechtesten Zustandes – Gehen Sie:

0: Normal
1: Annähernd normal – ein wenig langsamer
2: Langsam aber völlig eigenständig
3: Brauche Unterstützung oder Gehhilfe
4: Gehunfähig.

*Frage 2*. Beeinträchtigen Ihre Gehstörungen Ihr tägliches Leben, sowie Ihre Unabhängigkeit?

0: Überhaupt nicht
1: Nur geringfügig
2: Mässig
3: Stark
4: Gehunfähig.

*Frage 3*. Haben Sie das Gefühl, Ihre Füsse würden am Boden kleben während Sie gehen, sich drehen oder versuchen loszugehen (Einfrieren)?

0: Nie
1: Sehr selten – ungefähr einmal pro Monat
2: Selten – ungefähr einmal pro Woche
3: Häufig – ungefähr einmal pro Tag
4: Immer – jedes Mal, wenn ich gehe.

*Frage 4*. Wie lang dauert Ihr längster Vorfall des Einfrierens?

0: Kam noch nie vor
1: 1-2 Sek.
2: 3–10 Sek.
3: 11–30 Sek.
4: Gehunfähig für mehr als 30 Sek.

*Frage 5*. Wie lang dauert Ihr typisches Zögern beim losgehen (Einfrieren des Gangs beim ersten Schritt)?

0: keines
1: dauert länger als 1 Sek. loszugehen
2: dauert länger als 3 Sek. loszugehen
3: dauert länger als 10 Sek. loszugehen
4: dauert länger als 30 Sek. loszugehen.

*Frage 6*. Wie lang dauert Ihr typisches Zögern beim sich Drehen: (Einfrieren bei der Drehung)?

0: keines
1: Fortsetzung der Drehung in 1-2 Sek.
2: Fortsetzung der Drehung in 3–10 Sek.
3: Fortsetzung der Drehung in 11–30 Sek.
4: Unfähigkeit, Drehung fortzusetzen nach mehr als 30 Sek.
